# Efficacy of yogurt drink with added plant stanol esters (Benecol^®^, Colanta) in reducing total and LDL cholesterol in subjects with moderate hypercholesterolemia: a randomized placebo-controlled crossover trial NCT01461798

**DOI:** 10.1186/1476-511X-13-125

**Published:** 2014-08-06

**Authors:** Elsa M Vásquez-Trespalacios, Johanna Romero-Palacio

**Affiliations:** Epidemiology and Biostatistics Division, Medical School, Universidad CES, Medellín, Colombia

**Keywords:** Hypercholesterolemia, Low-density lipoproteins, Cardiovascular diseases, Plant stanol ester, Crossover

## Abstract

**Background:**

Cardiovascular diseases have become the leading cause of death from chronic diseases in the world. Main risk factors include hypercholesterolemia, which is caused in most cases by a high saturated fat diet. Plant stanol esters partly block cholesterol absorption in the digestive tract and thereby reduce total cholesterol and low-density lipoprotein (LDL) cholesterol serum levels. Based on epidemiological data, a 10 percent reduction of LDL cholesterol leads to a 20 percent decrease in the coronary heart disease risk throughout life. The aim of this study was to evaluate the efficacy of yogurt drink with added plant stanol esters (Benecol^®^ yogurt drink) in higher doses than the typically used (2g/d stanols), in lowering blood lipids in moderately hypercholesterolemic subjects.

**Methods:**

A randomized double-blind crossover, placebo-controlled study in moderately hypercholesterolemic subjects (n = 40) aged between 20 and 50 years old.

**Results:**

Yogurt drink with added plant stanols (4 g) as esters (Benecol^®^, Colanta) consumption compared to regular yogurt drink caused a statistically significant decrease in total cholesterol and low density lipoprotein cholesterol by 7.2% and 10.3%. During the two periods and compared to controls, high-density lipoprotein cholesterol and triglycerides were not significantly different.

**Conclusions:**

Yogurt drink with an active ingredient in Benecol^®^, plant stanol esters, reduced total cholesterol and LDL cholesterol in moderately hypercholesterolemic subjects.

**Trial registration:**

NCT01461798.

## Background

Phytosterols and their saturated form phytostanols, are naturally found in fruits, vegetables, nuts, seeds, legumes, vegetable oils, and from other sources [1–4]. Structurally, they are similar to cholesterol, but their absorption rate is much lower. Foods with added plant stanol esters, which could be cholesterol-lowering functional foods, provide the consumer an opportunity for cardiovascular disease prevention [5].

The cholesterol-lowering mechanism of plant sterols and plant stanols lies in the reduced intestinal absorption due to a replacement of cholesterol in intestinal micelles [6]. The structural similarities to cholesterol are responsible for both hypocholesterolemic effects and also lowered absorption at the intestinal level [7, 8]. Tests indicate an intestinal effect that decreases cholesterol absorption [9–11], which gains an important place in strategies to reduce cardiovascular risk [12].

Several clinical studies show that plant stanol esters are effective for lowering plasma total and LDL-cholesterol (LDL-C) levels [13–15] and this reduction occurs within two to three weeks after including foods with added dietary plant stanol esters [16]. With a regular consumption, the effect remains constant at least up to a year [13]. A meta-analysis of both plant stanol and plant sterol ester studies yielded on average an 8.8% reduction in LDL-cholesterol for a mean daily dose of 2.15 g phytosterols [17]. Such a reduction in LDL-C is beneficial for cardiovascular health. Based on epidemiological data, a 10 percent reduction of LDL cholesterol leads to a 20 percent decrease in the coronary heart disease risk throughout life [18].

A review of clinical evidence supports the notion that higher doses than currently recommended of plant stanol esters could have a dose-effect relationship between consumption and reduced serum LDL cholesterol concentrations [19]. A meta-analysis of 113 publications suggests that an ingestion of more than 2 g/day of plant stanols is associated with an additional LDL-C reduction compared to the lower doses [20]. The recent European Food Safety Authority Panel in Dietetic Products, Nutrition, and Allergies concluded that plant stanol esters at a daily intake of three grams (range 2.7 g to 3.3 g) lowered LDL-cholesterol by 11.4 percent [21].

Here, we evaluated the efficacy of a yogurt drink with added plant stanols as esters (in Benecol^®^, Colanta) in higher than the typically recommended dose in reducing total and LDL cholesterol in Colombian subjects with moderate hypercholesterolemia. This is the first study that investigates the efficacy of four grams of plant stanols as ester incorporated in a yogurt drink administered in two portions, daily.

## Results

### Clinical characteristics of subjects

We evaluated forty subjects, average age of 37.9 years, of whom the majority were women (n = 30, 75%). The average weight was 65.6 kg (62.9 kg for women and 73.7 kg for men) and is shown in Table 1. No differences in baseline variables according to the treatment sequence were found.

**Table 1:** **Subject characteristics at baseline**

Parameter		All subjects
Age (years)	Mean	37.9
	SD	8.5
	Range	25-50
Weight (kg)	Mean	65.6
	SD	10.3
	Range	48-91
Height (m)	Mean	1.62
	SD	0.08
	Range	1.45-1.80
BMI (kg/m^2^)	Mean	25.0
	SD	3.4
	Range	18.5-33.3
Sex	Female	30 (75.0%)
	Male	10 (25.0%)
Body mass index categories	18.5-24.9	20 (50.0%)
	25.0-29.9	17 (42.5%)
	30.0-34.9	3 (7.5%)

All subjects in the study were compliant to the intervention, with a consumption percentage reaching 80 percent, which was verified by counting the unopened and empty containers before each new delivery. During the entire follow-up period, none of the subjects suffered serious adverse events or other qualifying events. Four percent of the volunteers indicated that yogurt drink consumption improved their intestinal transit.

All subjects underwent tests for liver, kidney function, and blood count to check health conditions. None of the subjects suffered from any disease or disorder than moderate hypercholesterolemia. Laboratory tests confirmed that there was no abnormality. There was no change in these laboratory analyses regarding the order of the intervention, Table 2.

**Table 2:** **Safety laboratory data before and after intervention (week 10)**

Safety parameter		Before intervention (n = 40)	After intervention (n = 40)
Serum (S) ALAT IU/l	Median IQR		
		20.0	21.0
		16.0-32.3	16.3-30.5
S ASAT IU/l	Median IQR		
		23.0	23.0
		19.3-26.8	19.3-25.0
S Crea mg/dl	Median IQR		
		0.90	0.88
		0.81-1.00	0.77-1.01
S AFOS U/l	Median IQR		
		64.0	66.0
		50.3-82.3	54.3-79.8
Blood (B)Hb g/dl	Median IQR		
		14.1	14.3
		13.5-15.4	13.6-15.3
B Hkr%	Median IQR		
		40.8	41.4
		39.5-44.0	39.7-44.1
B Lymp%	Median IQR		
		37.1	40.5
		32.5-42.1	34.9-45.6
B Tromb mm^3^	Median IQR		
		279.5	284.5
		223.0-318.5	228.3-331.5

Differences in measurements of the above mentioned tests, did not exceed the limits established in the laboratory reference values. The limits of biological variability according to Westgard states for ALT changes an interindividual variation coefficient of 18 percent, for the AST of 11.9 percent, for serum creatinine 6 percent, alkaline phosphatase 6.4 percent, hemoglobin and hematocrit 2.8 percent, lymphocytes 10.4 percent, and platelet count of 9.1 percent. These values were not overcome by measuring the changes in subjects [22].

### Effect of treatments on blood lipids

**Figure 1:**
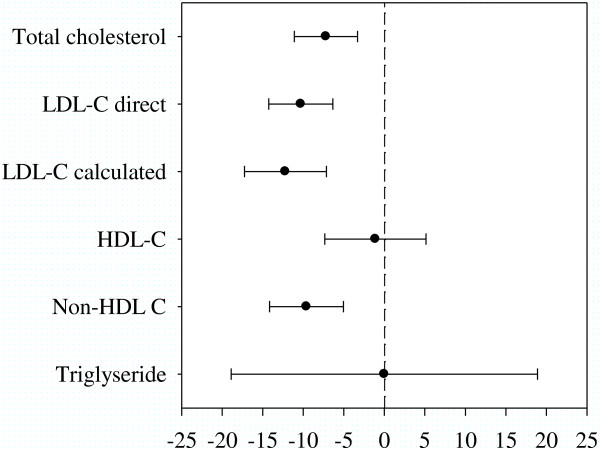
**Difference between plant stanol ester and placebo treatments for serum lipids (% change from first-period baseline).**

Following a four-week consumption of Benecol^®^ yogurt drink, total cholesterol was reduced by 15.6 mg/dl and proportionally by 7.2 percent compared to placebo drink (p = 0.001, Tables 3 and 4) The reduction of LDL cholesterol, when measured directly or calculated by the Friedwald equation was higher. LDL cholesterol was higher during the intervention period mounting to 10.3 percent and 12.2 percent, respectively (p < 0.001, Table 3, Figure 1).

**Table 3:** **Serum lipids (mg/dl) at the end of 4 weeks Benecol** ^**®**^ **and at the end of placebo treatment**

	Benecol		Placebo		Benecol^®^ vs. placebo		p
	Mean	(95% CI)	Mean	(95% CI)	Mean	(95% CI)	
Total cholesterol (mg/dL)	205.5	(197.9 – 213.2)	221.1	(212.8 – 229.4)	-15,6	(-24.2 to -6.9)	0.001
HDL cholesterol (mg/dL)	49.6	(45.8 - 53,4)	49.5	(45.1 – 53.9)	0.1	(-4.0 to 4.3)	0.95
LDL cholesterol measured (mg/dL)	108.7	(102.3 – 115.0)	120.7	(113.5 – 127.8)	-12.0	(-16.5 to -7.5)	<0.001
LDL cholesterol calculated (mg/dL)	125.8	(117.9 – 133.7)	142.2	(134.0 – 150.4)	-16.4	(-23.4 to -9.3)	<0.001
Non-HDL cholesterol (mg/dL)	155.9	(147.3 - 164.5)	171.6	(162.5 – 180.7)	-15.7	(-23.0 to -8.3)	<0.001
TG^3^ (mg/dL)	176.4	(137.2 – 215.7)	168.8	(138.7 – 198.9)	7.6	(-23.9 to 39.1)	0.63

^3^The distribution of TG was skew to the right.

After logarithmic transformation, the geometric means were 146.2 in Benecol^®^ and 149.4 in placebo (p = 0.76).

**Table 4:** **Percentage changes in serum lipids from first-period baseline to the end of 4 weeks Benecol and to the end of placebo treatment and differences in responses between treatments**

	Benecol		Placebo		Benecol^®^ vs. placebo		p ^1^
	Mean	(95% CI)	Mean	(95% CI)	Mean	(95% CI)	
Total cholesterol	-7.7	(-10.5 to -4.9)	-0,5	(-4.0 to 3.1)	-7.2	(-11.1 to -3.3)	0.001
HDL cholesterol	-1,1	(-4.8 to 2.6)	-0,1	(-6.9 to 6.8)	-1.1	(-7.3 to 5.2)	0.735
LDL cholesterol measured	-10,4	(-14.0 to -6.9)	-0,1	(-5.1 to 4.9)	-10.3	(-14.3 to -6.4)	<0.001
LDL cholesterol calculated	-11,5	(-16.6 to -6.4)	0,7	(-5.6 to 7.0)	-12.2	(-17.3 to -7.2)	<0.001
Non-HDL cholesterol	-9,3	(-12.9 to -5.8)	0,2	(-4.5 to 5.0)	-9.6	(-14.1 to -5.0)	<0.001
TG^3^	18,2	(1.6 to 34.8)	18,2	(3.2 to 33.3)	0.0	(-18.9 to 18.9)	0.999

^1^Test.

After logarithmic transformation the geometric means were 146.2 in Benecol^®^ and 149.4 in placebo (p = 0.76).

No statistically significant change for either HDL cholesterol (p = 0.735) or triglycerides (TG) (p = 0.999) were detected (Table 4, Figure 1). No period or carry-over effects were detected for any of the lipid outcome variables (all NS).

A significant difference existed between men and women’s baseline HDL cholesterol, non-HDL cholesterol, and in TG (data not shown). In a cross-over study, these differences are not important because the analysis is based on within-subject comparison, not on the between-subjects comparisons. Between BMI groups, the differences were non-significant with respect to all serum lipid variables. Being overweight (BMI ≥ 25 kg/m^2^) was observed in 14 women (46.7%) and in six men (60.0%) without significant difference (p = 0.465, Chi-squared test). All interactions between gender/BMI were nos significant (p > 0.20 in all analyses).

In the sub-group evaluation of the impact of gender and BMI status (overweight vs. normal weight) for total- and LDL-cholesterol-lowering by plant stanols, subjects with a BMI of less than 25 kg/m2 total cholesterol reduction during Benecol^®^ treatment (vs. placebo) was somewhat larger (18.5 mg/dl, 8.6%) compared to those whose BMI was greater than or equal to 25 (12.6 mg/dl, 5.8%). LDL reduction showed the opposite trend (11.3 mg/dl, 9.6%) and (12.8 mg/dl, 11.0%), in BMI of less than 25 and BMI greater than or equal to 25, respectively. In men, the cholesterol reduction during Benecol^®^ treatment (vs. placebo) was (16.9 mg/dl, 7.2%) and in women (15.4 mg/dl, 7.4%). LDL-cholesterol reduction was 14.2 mg/dl (10.8%) and 11.8 mg/dl (10.7%), in men and women, respectively.

## Discussion

We showed that after four weeks of consuming Benecol^®^ yogurt drinks with four grams of added plant stanols, a decrease in total cholesterol by 7.2 percent occurred and in LDL-cholesterol by 10.3 percent.

These results are in line to those reported previously, when determining that the consumption of plant sterols decreases serum total cholesterol levels and LDL-cholesterol values by 7 to 10 percent [23–25]. The mechanism by which this effect is achieved is related to reduced cholesterol absorption of both dietary and biliary origin [26, 27]. Studies in rat models confirm that stanols are absorbed poorly [28]. Evidence suggests that intake of the recommended (2 g/day) or higher dose of plant stanols produces neither serious adverse effects nor health risks of concern, but provides potential benefits for cardiovascular health through reduced serum cholesterol levels [26].

The higher dose used in this study did not provide an enhanced effect. The time of intake was reported to be ideal for inducing the physiological changes to allow plant stanols to compete over cholesterol absorption [29]. However, the subjects might not have consumed the yogurt drink immediately after a meal and thus the liquid nature of the food product might have influenced the cholesterol-lowering efficacy. This may be the case as in the previous studies using yogurt drinks as the cholesterol-lowering efficacy is achieved by smaller doses of plant stanols. In the study by Algorta-Pineda and co-workers, a 10.3 percent reduction in LDL-cholesterol and in the study by Krienginyos, a 13.5 percent reduction was achieved by two grams of plant stanols in a yogurt drink [30, 31]. Interestingly, Seppo et al. administered the yogurt drink under supervision in a café and an 11 percent reduction in LDL-cholesterol was detected, while only a 3.2 percent reduction was achieved when the consumption of the test product took place unsupervised [25].

LDL-cholesterol-lowering obtained with plant stanol ester consumption is important because it exceeds the effect that can be reached by doubling the dose of any kind of statin alone could reach by approximately six percent. Combining statins with plant stanols in subjects requiring further reduction of cholesterol may be effective and safer for the patient rather than doubling the dose of statins [27].

Sterols or stanols have no effect on cholesterol bound to high-density lipoprotein (HDL) and triglycerides, a result that we also observed in this study as no significant changes were obtained in HDL cholesterol and triglycerides measurements [32].

In hypertriglyceridemic subjects, consumption of plant stanols decreases serum triacylglycerols (>2.3 mmol/L; p = 0.009) [33]. In this study, changes in triglycerides were not significant in subjects without elevated triglyceride levels.

Plasma phytosterols, in addition to lowering lipid levels, are markers of a healthy diet and a lower cardiometabolic risk [34].

So far, the only contraindication in the consumption of these compounds is the presence of sitosterolemia; an uncommon metabolic defect as is, characterized by an intestinal hyperabsorption of all sterols consumed in ordinary food, which is associated with premature atherosclerosis [35].

According to epidemiological data, a 10 percent reduction of LDL cholesterol leads to a 20 percent decrease in the coronary heart disease risk throughout life [18].

## Conclusions

Yogurt drink with added plant stanol esters reduced total cholesterol and LDL cholesterol in in subjects with moderate hypercholesterolemia.

## Methods

### Subjects

Volunteers were recruited and were people working or living in the neighborhood next to the laboratory where tests were performed. Before inclusion, blood samples were drawn to choose subjects with mild hypercholesterolemia (serum total cholesterol of 5.2 to 7.5 mmol/l (205-290 mg/dl)). Subjects with lipid-lowering medication or other drugs that significantly affect lipid values, diabetes type I or II, severe obesity (BMI greater than 35 kg/m^2^), fasting serum triglycerides > 4.0 mmol/l, liver, or kidney disorder according to medical history, history of coronary revascularization, percutaneous transluminal coronary angioplasty within six months prior to screening, history of temporary ischemic attack or stroke within six months prior to screening, history of cancer or other malignant disease in the last five years, consumption of more than 15 dosages of alcohol/week, pregnant or lactating, Benecol^®^ consumption in their diet, or other plant stanol or sterol enriched products 30 days before visit 2 (Week 1), severe lactose intolerance, milk allergy, or any other form of intolerance to the ingredients of the test products, celiac disease were excluded.

This study was approved by the Institutional Ethics Committee at Universidad CES, Medellín, Colombia and all subjects gave informed consent.

### Study design

**Figure 2:**
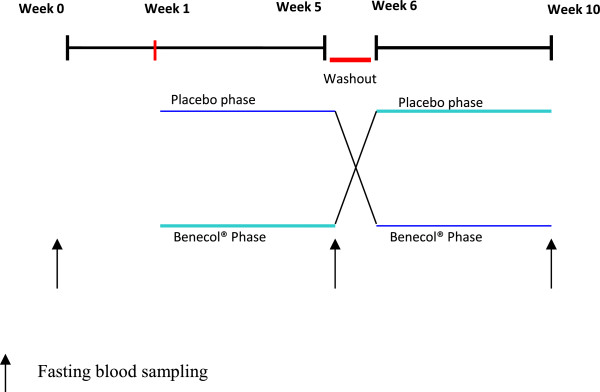
**Intervention scheme.**

This study used a double blind, placebo-controlled and randomized crossover study with two treatment periods lasting four weeks each, separated by a one-week wash out period (Figure 2). 60 moderately hypercholesterolemic volunteers were invited to participate and 40 of them met the inclusion criteria. The subjects were randomized and one group received the Benecol^®^ yogurt drink and the other group consumed placebo yogurt during four weeks (20 subjects per group). After a one-week wash-out period, the subjects who started with Benecol yogurt drink received the placebo yogurt drink and the subjects starting with placebo drink received Benecol yogurt drink for the next four weeks. Randomization of subjects was performed in (EPIDAT) version 3.1. Randomization, inclusion, and allocation of subjects to each group were made by the principal investigators.

The producers of the test product (COLANTA, Medellín, Colombia) were the only who knew the allocated intervention and both researchers and study participants were blind of this assignment, which was revealed once the study was completed. During the study, subjects were asked to keep their habitual diet and physical activity pattern unchanged. There was no other dietary intervention during the study except the consumption of the yogurt drink.

### Intervention

Blood sample was drawn at week zero for the assessment of the lipid profile and these results found that 40 subjects met the inclusion criteria. At week 1, a new blood sample was taken and this time subjects underwent tests to check liver and kidney function to meet the basic health conditions, after these results, subjects were allocated to consume placebo yogurt drink or Benecol^®^ yogurt drink (200 ml of yogurt a day as part of main meals, corresponding to 4 g of plant stanols as esters, daily) during four weeks. At the beginning of week 5, a new sample of blood from subjects was taken for determining lipid profile including total cholesterol LDL-cholesterol, triglycerides, and HDL cholesterol, subjects had a week off (washout period), and at week 6, groups were changed, (those consuming placebo yogurt were changed to Benecol yogurt drink and *vice versa*). At the end of week ten, a blood sample was drawn to verify that subject’s baseline liver and renal function were preserved and to measure changes in lipid levels (Figure 2).

### Yogurt drinks

The two study drinkable yogurts were produced by the dairy cooperative COLANTA (Medellin, Colombia) under controlled conditions, established in the production area, packed in a white package and labeled at the top with an expiration date and indicative of sample 1 and sample 2 to maintain the blinding setup.

Sample 1: Placebo Yogurt drink, 2 pots/day.

Sample 2: Benecol^®^ Yogurt drink with 2 g of plant stanols as esters per 100 ml, 2 pots/day.

Nutrient composition per 100 ml of the yogurt drink:

- Energy 54 kcal
- Fat 1.5 g
- Protein 2.8 g
- Carbohydrates 7.3 g
- Plant stanols 2.0 g

Supply of yogurt drinks was conducted between July to September 2012, subjects were given detailed instructions on how to use the test products. Yogurt drinks were produced once a week and delivered to the subjects. The study participants were advised to consume the yogurt drink with meals.

### Compliance

Compliance was assessed by interviewing subjects and by recording yogurt drink consumption. The subjects returned both unopened and empty consumed packages to the principal investigator which allowed determining the percentage of servings of yogurt drinks consumed by each subject at the scheduled time. Non-compliance to the study protocol was defined as less than 80% of consumption of the test yogurt drink.

### Safety

Some variables of interest were assessed in order to ensure the safety of volunteers; measurement of alanine aminotransferase (ALT), aspartate aminotransferase (AST), creatinine, alkaline phosphatase, hemoglobin, hematocrit, lymphocytes, platelet count was performed at first week to volunteers entry to the study; i.e., before starting the intervention and at the end of week ten.

### Analytical methods

Fasting blood samples were taken by venipuncture at baseline, week 5 and week 10. All venipuncture were generally carried out by the same person, at the same location. Subjects were asked to fast for at least eight hours before blood samples were taken. ALT, AST, FA, creatinine, LDL cholesterol and HDL cholesterol were assessed through an enzymatic technique (Biosystem^®^) United States. LDL-C was also calculated by Friedewald’s equation. Total cholesterol and triglycerides were assessed through a colorimetric technique (Biosystem^®^).

### Statistical analysis

Sample size calculations were based on the following assumptions. It was assumed that the effect of Benecol^®^ (vs. placebo) on the change in LDL cholesterol is between -8% and -12% and SD of within-subjects changes is 15% during both interventions. A sample size of 38 subjects will have 90% power to detect the 8% difference between Benecol^®^ and placebo treatments statistically significant with a 0.05 two-sided significance level. The differences of 10% and 12% between Benecol^®^ and placebo would require smaller sample sizes, 25 and 17 subjects, respectively.

Baseline characteristics for subjects in the two treatment sequences were compared using Chi-squared test for categorical variables, Mann-Whitney U test for laboratory safety variables and t-test for independent samples for other continuous variables.

Serum total cholesterol and LDL-cholesterol (both direct measured and calculated by Friedewald equation) were the primary outcome variables. Serum HDL-cholesterol, non-HDL cholesterol, and triglycerides (TG) were the secondary outcome variables. The primary and secondary variables were evaluated after the run-in period (before the first treatment period) referred to as the “first-period baseline” and at the end of each treatment period, referred to as “end of treatment”.

For all serum lipids both absolute values (mg/dl) at the end of treatments and percentage change (% change) from first-period baseline were analyzed. Repeated measures analysis of variance (ANOVA) for cross-over designs was used to test the effects of treatment, period, and carry-over. Although there were no significant period or carry-over effects for lipid variables expressed as absolute value or percentage change, the order of treatments was included in all models. The results are given as means with 95% confidence intervals. The distribution of serum triglyceride (mg/dl) was skew to the right and the analysis was repeated using the logarithmically transformed values. Statistical analyses were performed according to the intention to treat. All tests were two-sided and a p < 0.05 was considered to indicate a significant difference. Statistical analyses were performed using IBM SPSS Statistics (version 22.0, Armonk, NY: IBM Corp.).

## Authors’ information

Elsa Maria Vasquez-Trespalacios. Biologist, M.Sc. in Epidemiology. Associate researcher in Epidemiology and Biostatistics Division. Universidad CES. Medellín, Colombia.

Johanna Romero Palacio. Microbiologist, M.Sc. in Epidemiology. Associate researcher in Epidemiology and Biostatistics Division, Universidad CES. Medellín, Colombia.

## Abbreviations

HDL: High-density lipoprotein
LDL: Low-density lipoprotein
ALT: Alanine transaminase
AST: Aspartate transaminase
BMI: Body mass index
TG: Triglyerides.
